# Analysis of catalyst surface wetting: the early stage of epitaxial germanium nanowire growth

**DOI:** 10.3762/bjnano.11.121

**Published:** 2020-09-09

**Authors:** Owen C Ernst, Felix Lange, David Uebel, Thomas Teubner, Torsten Boeck

**Affiliations:** 1 Leibniz-Institut für Kristallzüchtung, Max-Born-Straße 2, 12489 Berlin, Germany

**Keywords:** dewetting, germanium, interfacial energy, Laplace pressure, nanostructure, nanowire, Ostwald ripening, wetting layer

## Abstract

The dewetting process is crucial for several applications in nanotechnology. Even though not all dewetting phenomena are fully understood yet, especially regarding metallic fluids, it is clear that the formation of nanometre-sized particles, droplets, and clusters as well as their movement are strongly linked to their wetting behaviour. For this reason, the thermodynamic stability of thin metal layers (0.1–100 nm) with respect to their free energy is examined here. The decisive factor for the theoretical considerations is the interfacial energy. In order to achieve a better understanding of the interfacial interactions, three different models for estimating the interfacial energy are presented here: (i) fully theoretical, (ii) empirical, and (iii) semi-empirical models. The formation of nanometre-sized gold particles on silicon and silicon oxide substrates is investigated in detail. In addition, the strengths and weaknesses of the three models are elucidated, the different substrates used are compared, and the possibility to further process the obtained particles as nanocatalysts is verified. The importance of a persistent thin communication wetting layer between the particles and its effects on particle size and number is also clarified here. In particular, the intrinsic reduction of the Laplace pressure of the system due to material re-evaporation and Ostwald ripening describes the theoretically predicted and experimentally obtained results. Thus, dewetting phenomena of thin metal layers can be used to manufacture nanostructured devices. From this point of view, the application of gold droplets as catalysts to grow germanium nanowires on different substrates is described.

## Introduction

Wetting phenomena as well as the formation and movement of droplets are essential for numerous applications. Surface treatment, for example, modifies the wetting behaviour of active fluids on composite materials or porous media to increase the efficiency and selectivity of catalytic processes [1]. Droplet-based microfluidics, including on-chip and off-chip incubation in immiscible phases, even developed into an independent field of science [2]. This shows that understanding wetting phenomena is crucial for a variety of industrial processes and research fields. In microtechnology, the dispersion of organic photoresists on substrates is indispensable for lithographic top-down microstructuring, yielding highly functional intermediate products for further processing [3–4]. The presence of droplets and dewetting phenomena are not only observed in aqueous and organic systems, but also in inorganic systems, such as liquid metals [5] and ultrathin layers [6]. The formation of metallic nanodroplets can be beneficial when intentionally used (e.g., to create strongly localized heat sources [7–8]), but it can also be disruptive (e.g., when ultrathin copper layers collapse on titanium nitride, damaging electronic devices [9–10]). However, in thin film applications these phenomena are rarely attributed to the dewetting process, since the name of the resulting structures are nanoparticles or clusters but rarely droplets. Nevertheless, the origin of these structures from fluid-like states offers the opportunity for novel bottom-up techniques to produce precursor materials for functional materials, such as chalcopyrites [11], or precursors for complex structures, such as nanowires [12]. Silicon, germanium and silicon oxide nanowires, for example, can be formed on different substrates by using metal catalysts in the form of tin, indium or gold nanodroplets [13–15]. Such nanometre-sized one-dimensional materials are, therefore, promising for gate-all-around architectures [16–17], which are very attractive for future low-power field-effect transistors (FETs) [18] and thermoelectrics [19–21].

The formation of nanodroplets leads to various outcomes, depending on the combination of substrate and droplet material. Thus, the interaction between the catalytic nanodroplets and the substrate surface must be investigated in more detail to provide a better understanding and control over the process. In the present work, the surface diffusion processes were examined in order to understand and effectively control the formation of the nanostructures.

The wetting behaviour of gold deposited either on silicon or silicon oxide wafers was studied. The property of gold to form a layer, droplets, or particles on silicon or silicon oxide was theoretically described and experimentally demonstrated by ultrahigh vacuum physical vapour deposition (UHV−PVD). The theoretical models are based on a fundamental description of the dewetting phenomenon from an energetic point of view. Closer attention is given to the thermodynamic differences between gold and silicon or between gold and silicon oxide in terms of free energy and interfacial potentials. For this purpose, the interfacial potentials were calculated by using three different methods: an empirical approach, in which the wetting angle was taken into account (“WA model”); a completely theoretical approach, in which the interface was described purely by van der Waals interactions (“vW model”); and a semi-empirical approach, in which R. H. Ewing's considerations towards the interfacial energy between a solid metal and its melted form were applied to study the substrate–fluid interface between different materials (“AE model”). A more detailed description of the three methods can be found in the Experimental section. The resulting free energy leads to different nanodroplets, which changed their catalytic behaviour during nanowire growth.

## Results and Discussion

### Theoretical results

#### Gold on silicon oxide

**Figure 1:**
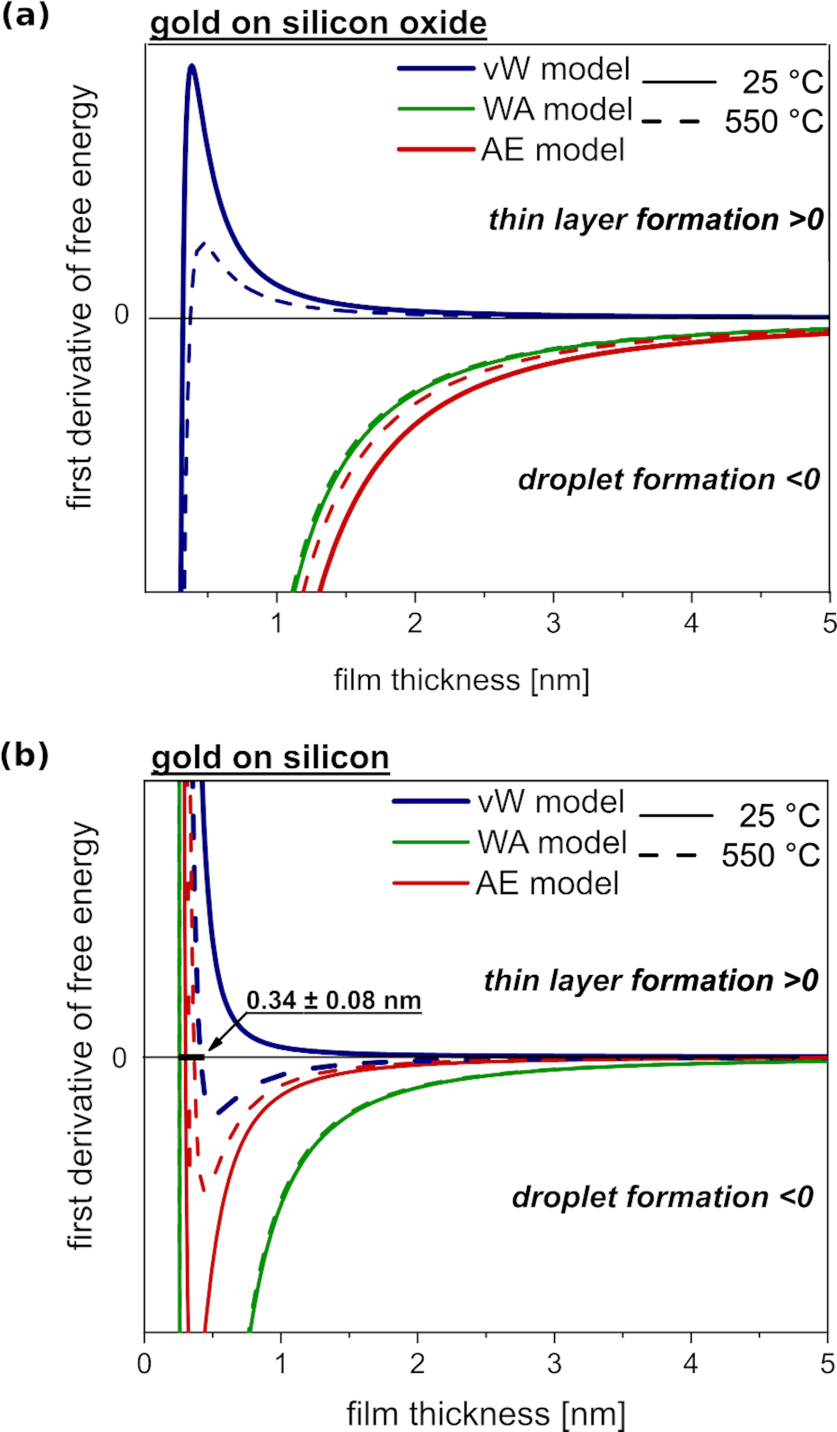
Derivative of the free energy as a function of the film thickness for (a) gold on silicon oxide and (b) gold on silicon at 25 °C (solid lines) and 550 °C (dashed lines). The calculations for the interfacial energy were made by using three different models: vW (blue), WA (green) and AE (red). Values above zero indicate a stable thin layer of gold, while values below zero indicate an unstable thin layer, which consequently results in the formation of droplets or particles.

Figure 1 shows the first derivative of the free energy with respect to the layer thickness. Two different types of wetting behaviour of gold on silicon oxide are predicted, depending on the model used. While the vW model has a zero-crossing in *d*_real_ = 0.38 nm, the other two models follow a classical dewetting mechanism: The function of the first derivative only assumes values below the abscissa and converges towards zero. This is a major distinction since values above zero leads to the formation of a continuous thin film, whereas values below zero promote the formation of droplets on the surface.

Since a gold monolayer has a nominal thickness of 0.32 nm, the maximum thickness of 0.38 nm, predicted in the vW model at 550 °C, exceeds the monolayer thickness and a second gold monolayer starts to form. Consequently, this model predicts the growth of a layer of gold on silicon oxide. In contrast to the vW model, the other two models predict droplet formation over the entire thickness range.

#### Gold on silicon

In contrast to gold on silicon oxide, gold on silicon (Figure 1b) shows a dependence of the free energy on the temperature. As the temperature rises, the first derivative of the free energy and its corresponding slope increase. Qualitatively, all three models predict the same behaviour: Gold forms droplets on silicon with a wetting layer between the droplets. The thickness of the wetting layer is approx. 0.28 ± 0.02 nm, at room temperature, and approx. 0.34 ± 0.08 nm at 550 °C, since the zero-crossing of the graphs occurs at these values. Assuming that a gold monolayer has a thickness 0.32 nm, one or two gold monolayers are stable on silicon, as long as the systems are in thermodynamic equilibrium.

### Experimental results

#### Gold on silicon oxide

**Figure 2:**
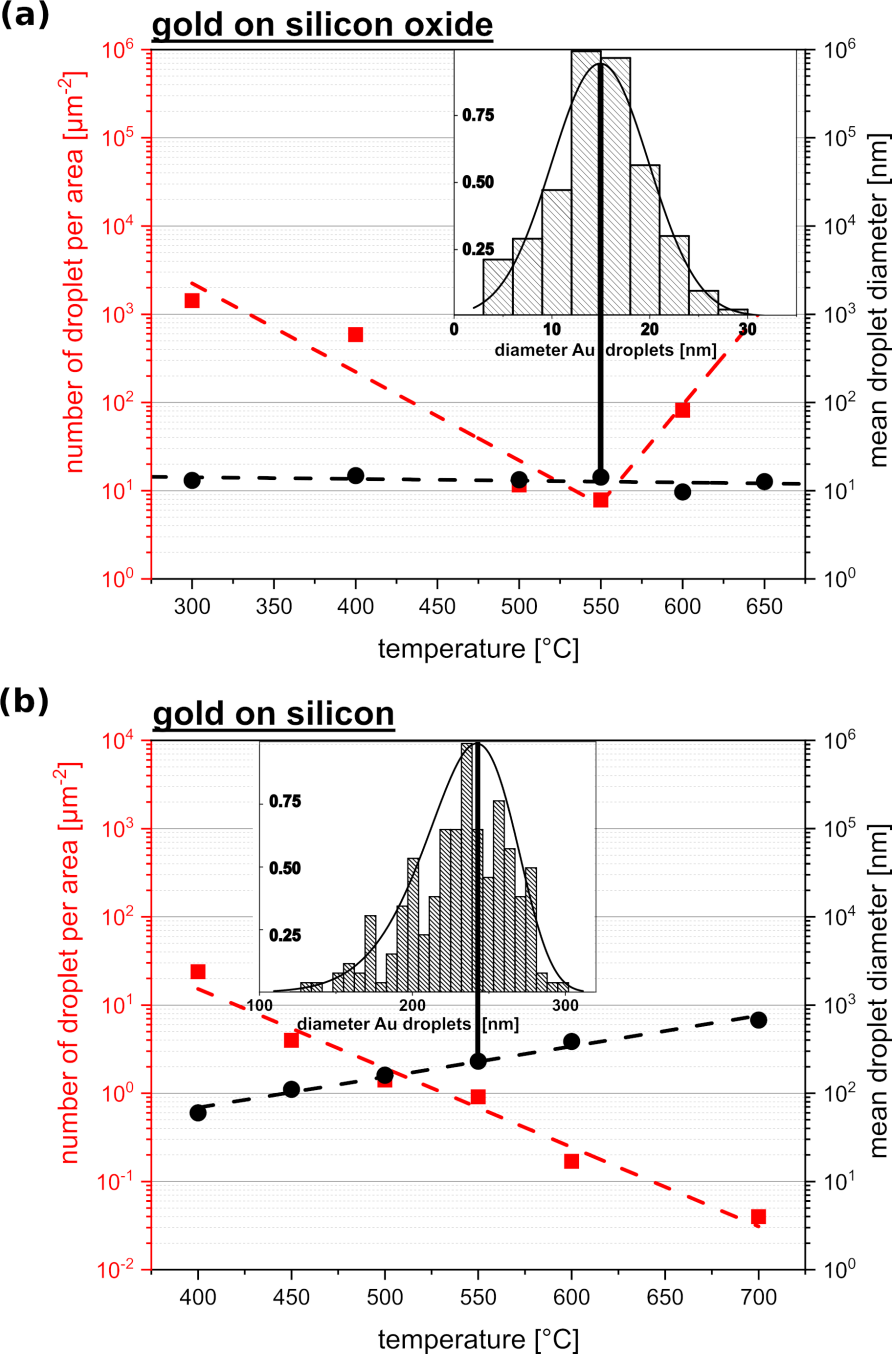
Behaviour of gold droplets on (a) silicon oxide and (b) silicon. The plots show the number of droplets per area (red squares, guide to the eye: dashed red line) and mean droplet diameter values (black dots, guide to the eye: dashed black line) as a function of the substrate temperature. The insets show histograms with the size distribution of droplet diameter values at 550 °C. For Au on SiO*_x_* the distribution is approximated by a Gaussian function. For Au on Si the distribution is given by a Lifshitz–Slyosov–Wagner (LSW) expression.

UHV−PVD-deposited gold droplets on SiO*_x_* are characterized in Figure 2a. The dependence of gold droplet mean diameter and the number of gold droplets per area on the substrate temperature is shown. The size distribution at 550 °C is also depicted (Figure 2, insets), which is representative of all distributions within the temperature range considered. The diameter of the gold droplets remains constant throughout, while the size distribution is described by a Gaussian distribution curve. The number of droplets per area as a function of the temperature follows two trends, which change at approximately 550 °C. At this point the number of droplets is minimal and this value increases away from this point.

#### Gold on silicon

**Figure 3:**
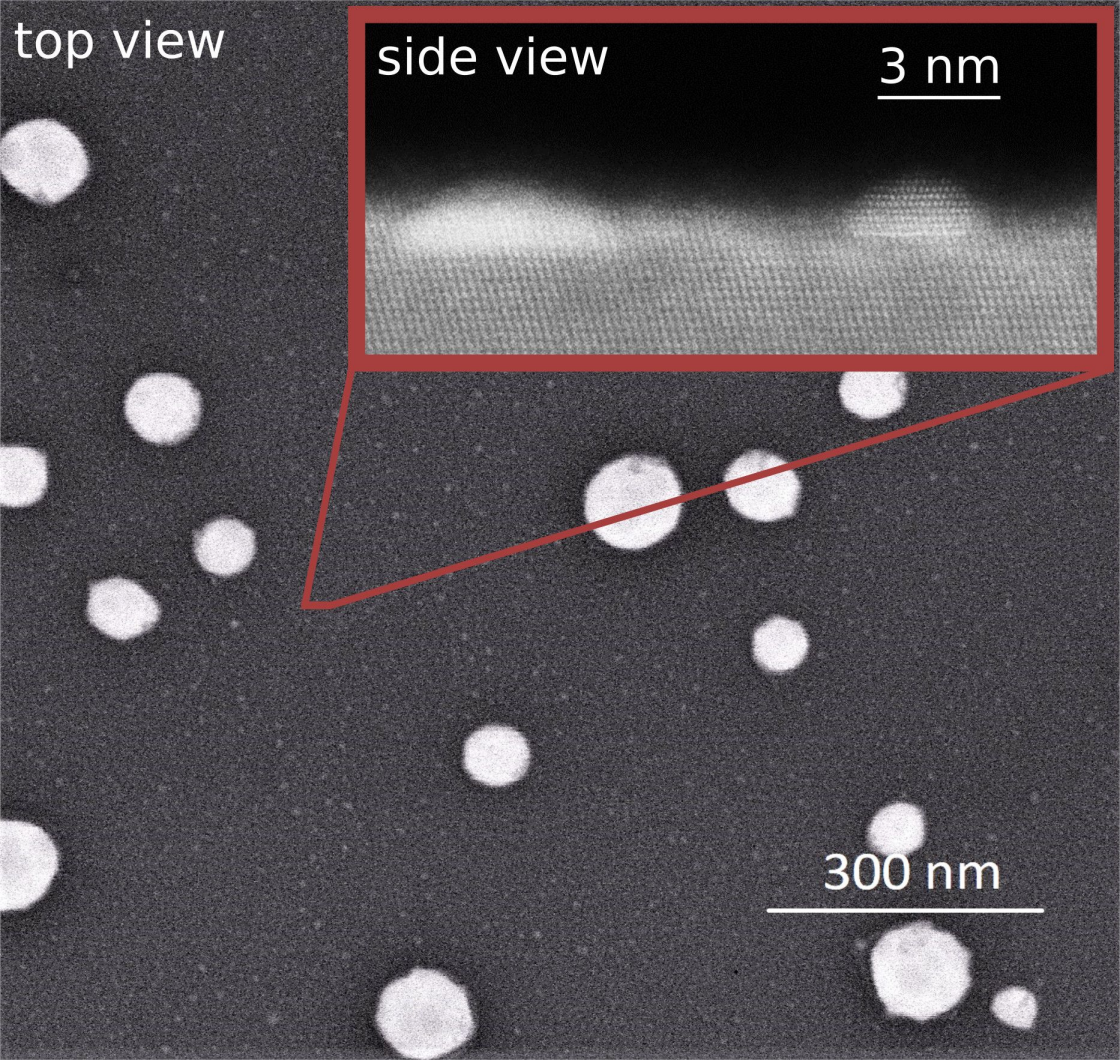
SEM image of gold on silicon after the system reached room temperature. The inset shows a TEM image, in which small gold clusters nucleated on the silicon surface between the droplets can be seen.

Figure 2b shows the results for gold droplet formation on Si(111). A decrease in droplet number and an increase in droplet diameter with increasing temperature can be visualized. The droplet diameter distribution is described by a LSW distribution. Figure 3 shows scanning electron microscopy (SEM) and transmission electron microscopy (TEM) images of gold particles formed on a silicon substrate at room temperature. Small gold clusters (<10 nm) are also seen between the droplets.

#### Growth of germanium nanowires

**Figure 4:**
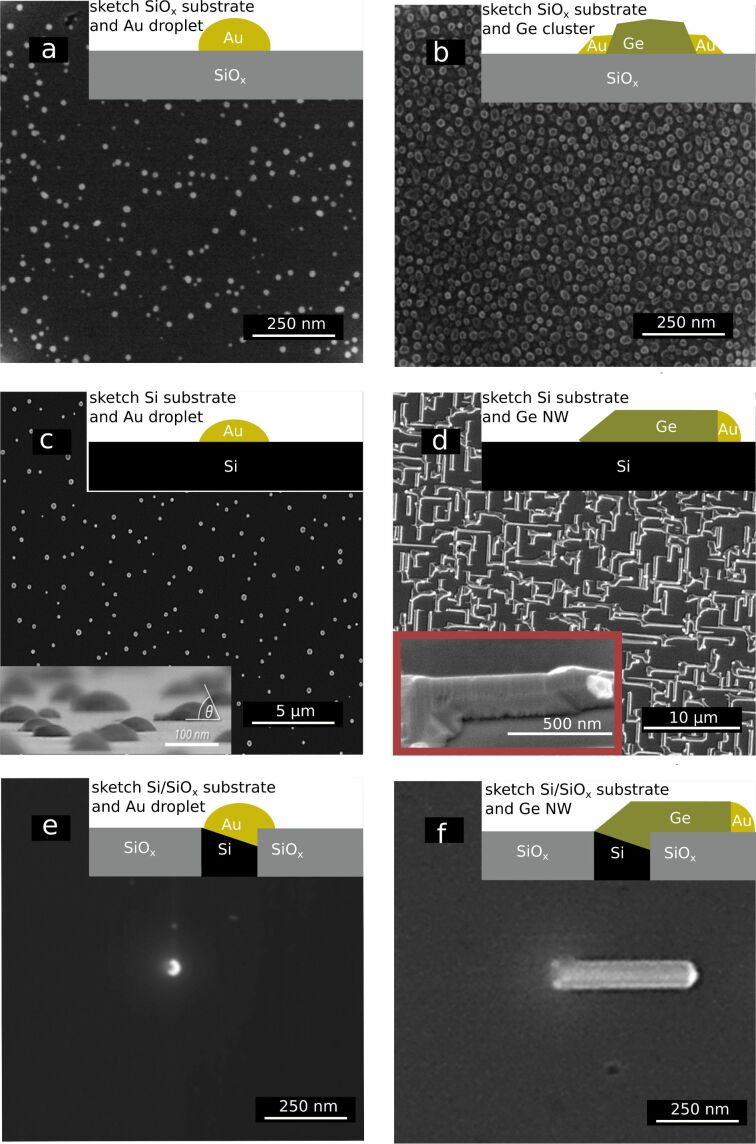
SEM images of the gold droplets and the results of the experiments to grow germanium nanowires. The insets at the upper right corner illustrate side views of the systems studied. (a) Top-view image of gold nanodroplets (approx. 15 nm) formed on silicon oxide substrate. (b) Image of a gold/silicon oxide sample after germanium deposition. Under these conditions, the gold droplets did not catalyse germanium nanowire growth. Instead, germanium clusters were formed. (c) Top-view image of gold nanodroplets (approx. 100 nm) formed on silicon. The inset at the lower left corner shows an image of an inclined sample with a drawn-in wetting angle θ. (d) Image of germanium 1D structures grown on a silicon substrate, which were formed via the catalysis of gold droplets during germanium deposition. The inset at the lower left corner shows an image of an inclined sample. The droplet at the top of the germanium nanowire is clearly visible. (e) Top-view image of a single gold nanodroplet at a silicon tip in a silicon oxide matrix. As it can be seen, the droplet sits at only one side of the silicon tip. (f) Image of the sample shown in (e) after germanium deposition. The gold droplet catalysed the growth of a germanium nanowire.

Figure 4 shows images of the resulting gold droplets on various substrates and the results after deposition of germanium on these samples. In Figure 4a, a silicon oxide substrate was used for gold deposition. In Figure 4b, germanium was deposited onto a silicon oxide sample containing gold droplets at a deposition rate of 0.005 nm·s^−1^ and at 500 °C. No 1D structures were observed; instead, random germanium clusters were formed throughout the sample surface. The results for gold and germanium deposition onto a silicon substrate are shown in Figure 4c and Figure 4d, respectively. In Figure 4c, the gold droplets formed on the Si substrate were larger than the ones formed on the SiO*_x_* substrate (Figure 4b). When germanium was deposited onto Au/Si substrates, germanium nanowires were grown. The in-plane nanowires started to grow at places where the gold droplets had formed previously. The inset shows gold at the top of the germanium nanowire, where continuous homoepitaxial growth was catalysed. A sample with a silicon nanotip [22] surrounded by a silicon oxide matrix is shown in Figure 4e and Figure 4f. A gold droplet is formed at the silicon tip during gold deposition (Figure 4e). After germanium deposition, the gold droplet at the silicon tip forms a single in-plane nanowire at the surface of the silicon oxide matrix (Figure 4f).

## Discussion

This work had three major aims and the first one was to compare between three different interfacial energy models and how they agreed with the experimental results (Table 1). The second aim was to investigate the wetting behaviour of gold when deposited onto silicon oxide or silicon substrates. The third and last aim was to verify and compare the behaviour of germanium when deposited onto substrates containing either gold and silicon oxide or gold and silicon.

**Table 1:** Comparison between three interfacial energy models (WA, vW, and AE) and their corresponding experimental results.

model (abbreviation)	WA	vW	AE

name	wetting angle	van der Waals	R. H. Ewing approach
formula for the interfacial energy	Equation 1	Equation 2	Equation 3
formula for the resulting free energy	Equation 4	Equation 5	Equation 6
approach	empirical	theoretical	semi-empirical
short description	Wetting angles between substrate and droplets are connected to the interfacial energy by the Young–Dupré equation.	The interfacial energy is related to the polarizabilities of the used materials. This can be estimated by the long- range contribution of the van der Waals energy.	The interfacial energy between a solid and a fluid consists of two parts: a solid part and a fluid part. These fractions can be estimated based on material parameters.
assuming nanodroplets of Au on SiO*_x_*	yes (in agreement with experimental results)	no (not in agreement with experimental results)	yes (in agreement with experimental results)
assuming a wetting layer of Au on SiO*_x_*	no (in agreement with experimental results)	yes (not in agreement with experimental results)	no (in agreement with experimental results)
assuming nanodroplets of Au on Si	yes (in agreement with experimental results)	yes (in agreement with experimental results)	yes (in agreement with experimental results)
assuming a wetting layer of Au on Si	yes (in agreement with experimental results)	yes (in agreement with experimental results)	yes (in agreement with experimental results)
strengths	high agreement with experimental results; simple	no empirical data needed	high agreement with experimental results; little susceptibility to faults
weaknesses	only available for systems with droplet formation; hysteresis effects	very prone to errors; only valid for well-known systems; fails for Au on SiO*_x_*	requires a high amount of data

### Gold on silicon oxide

The WA and AE models show that a thin gold layer on silicon oxide is unstable and, therefore, gold tends to form either droplets or particles on the surface (Figure 1). Only the fully theoretical interfacial energy vW model predicts a free energy, which leads to a stable thin gold layer with thicknesses above 0.38 nm. However, the experimental data, as shown in Figures 2–4, indicate that the vW model fails since gold droplets are formed on the silicon oxide surface. A deep insight into the characteristics and properties of the observed material is needed to perform calculations using van der Waals interactions. However, thermal silicon oxide has no defined crystallographic orientation and no defined surface chemistry, since different oxide types can occur. The vW model fails to provide a complete theoretical description of gold deposition onto silicon oxide. This happens since there is not enough information about the morphology and chemical heterogeneity of silicon oxide surfaces. Even for thermal silicon oxide surfaces, which are grown in a well-controlled manner, only approximate values are given in the literature [23]. In addition, the van der Waals energy between silicon oxide and gold is repulsive, which is unsuitable for a model that assumes two surfaces that are brought closer together. For such complex and repulsive systems, empirical or semi-empirical methods are more appropriate.

On silicon oxide, the number of droplets per area of gold decreases with increasing temperature until the latter reaches 550 °C. At higher temperature values, the number of droplets rises again. The mean droplet diameter remains constant over the entire temperature range. This effect can be explained by droplet re-evaporation. The vapour pressure of bulk gold at 550 °C is between 10^−11^ and 10^−13^ mbar [24] and it becomes even higher on highly curved surfaces, such as droplets. Small droplets are characterized by a high surface curvature, which leads to a high Laplace pressure. As the temperature rises, the Laplace pressure in the droplets increases, increasing the likelihood of re-evaporation as long as the pressure is not reduced by other effects, such as Ostwald ripening. Ostwald ripening requires the existence of a medium where the material can be transported. Since there is no wetting layer (according to the WA and EA models, Figure 1), the Laplace pressure cannot be relieved by Ostwald ripening. Nevertheless, an Ostwald ripening process could also take place through the gas phase, although this process is kinetically inhibited.

The re-evaporation process leads to a decrease in the number of droplets per area, while the droplet diameter values remain constant and the diameter distribution remains Gaussian, and no LSW distribution occurs as in Ostwald ripening processes [25–26]. This behaviour, derived from theoretical considerations, can be applied to the results in Figures 2−4. The increasing number of droplets above 550 °C can be attributed to a spinodal dewetting mechanism: Higher temperatures in the initial fluid layer lead to an increase in internal heat and faster movement of atoms. Thus, the system reaches a supercritical state, in which the layer thickness increasingly fluctuates until it finally destabilises. Therefore, higher temperatures result in higher numbers of droplets per area during dewetting.

### Gold on silicon

All three models show the same behaviour of droplet formation when gold is deposited onto a silicon substrate at 550 °C, which is confirmed experimentally and described in the literature [27]. This second system (i.e., gold on silicon) is simpler to model than the first system (i.e., gold on silicon oxide). Pure silicon wafers have a defined surface orientation with well-understood physical and chemical properties; therefore, even the fully theoretical vW model fits the real system behaviour. Nevertheless, the results for gold on silicon also vary due to the wide range of possible Hamaker constants, which are needed in the calculations. Hamaker constants correspond to the susceptibility of particles to an electric field of very small length scales generated by the particles themselves [28]. For this reason, these constants are used to determine energy and force values in van der Waals interactions. A more detailed description of this critical value can be found in the Experimental section. Hamaker constants can be obtained from the literature, measured, calculated by material constants (see Equation 7), or simulated by microscopic and macroscopic models. Since there are several ways to determine the Hamaker constants, this leads to a wide spectrum of different values. Furthermore, only the nonretarded interactions are considered; however, at higher thicknesses (>10 nm) the retarded interactions become more relevant. Nevertheless, all essential effects and values occur for thickness values below 1 nm. Since the Hamaker constant is a critical value for the models shown here, its wide range can lead to difficulties in obtaining reliable values for the free energy of a system, especially for the fully theoretical model, which focuses on van der Waals interactions. However, the theoretical predictions for gold on silicon fit very well to the observed experimental behaviour. The theoretical results predict a gold wetting layer of 0.34 ± 0.08 nm on a silicon substrate. As seen in Figure 2, the number of gold droplets per area decreases with increasing temperature, while the droplet diameter increases. This effect can be linked to the wetting layer, which functions as a communication layer between the droplets. This way it promotes material transport and accelerates the occurrence of certain events, such as Ostwald ripening. This argument is also supported by the fact that the droplet diameter values follows a LSW distribution, which is characteristic of Ostwald ripening processes. Nevertheless, it should be mentioned that thicker wetting layers can occur at high temperatures if the system deviates from its equilibrium state. It can be assumed that smaller droplets can be formed between larger droplets if the thickness of the wetting layer is forced to decrease during the cooling process, as shown in Figure 3. Very small gold clusters (<10 nm) are visible between the droplets. This indicates that the wetting layer collapses during cooling. It remains unclear whether the wetting layer completely disappears into these clusters or if it just becomes thinner or perforated. However, the TEM data do not show any evidence of a wetting layer at room temperature, since at this temperature only very small gold clusters are seen. This is in agreement with low-energy electron microscopy (LEEM) calculations, which predict a change in the surface recombination of gold on silicon formed during cooling [29]. The diffusion activation energy of the Ostwald ripening process, *E*_A_, can be calculated using the qualitative correlation


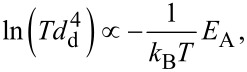


where *T* is the temperature, *d*_d_ is the mean droplet diameter, and *k*_B_ is the Boltzmann constant. For the data shown in Figure 2b, the value obtained for of *E*_A_ is 1.79 eV, which is in agreement with the values in the literature (1.56 eV). For gold on silicon oxide, the activation energy for surface diffusion cannot be calculated since the temperatures considered here are too low to cause a significant change in droplet size.

Moreover, it must be taken into account that Si(111) substrates were used for the experiments. Due to the anisotropy of silicon, the values shown here cannot be used quantitatively for other crystal orientations, since they have different surface and interfacial energies. However, the general qualitative trend was maintained when individual experiments were performed on Si(001) substrates. In the case of silicon oxide, due to its isotropy, these effects cannot be considered.

### Growth of germanium nanowires

The main reason why germanium nanowires grow on silicon but not on silicon oxide is related to the size of the gold droplets formed during dewetting. Since the VLS-induced nanowire growth is a catalytic process, a minimum amount of gold atoms inside the droplet is necessary to dissolve germanium inside the gold cluster. This minimum amount is exceeded only on silicon but not on silicon oxide. Even though there is evidence suggesting that it is possible to grow wires on silicon oxide substrates via VLS, either different types of silicon sources [30] or other methods to generate gold droplets were used, yielding significantly larger droplets than the ones used in this study [31]. These results also indicate a size effect.

If there are silicon and silicon oxide areas available on a substrate (as shown in Figure 4e and Figure 4f), gold places itself preferentially onto the silicon areas. Since a eutectic decomposition of gold and silicon takes place in bulk mixtures at 360 °C, it is safe to assume that there is a chemical affinity between gold and silicon. This chemical affinity is also indicated by the fact that up to 2 ppm of gold can be dissolved in bulk silicon at higher temperatures [32]. Therefore, the formation of a larger gold droplet on silicon is not an effect of the Laplace pressure (i.e., neither re-evaporation nor Ostwald ripening occur). It is just an effect of compound affinity. The droplet even etches the silicon to form a stable {111} plane, which is why the droplet seems to be placed just on one site of the silicon spot. The germanium nanowire then grows from the etched facet in ⟨110⟩ direction.

## Conclusion

A theoretical prediction of the behaviour of metal droplets on different surfaces was presented. For this purpose, thin gold layers of silicon oxide and silicon were prepared and investigated. When gold dewets from these surfaces, there is a formation of either gold droplets or particles on the surface. The theoretical description focuses on the determination of the interfacial energies between gold and silicon oxide and gold and silicon. Three models were presented: A fully theoretical model (vW model), a semi-empirical model (AE model), and a fully empirical model (WA model). The interfacial energies are needed in order to calculate the free energy and, more precisely, the first derivative of the free energy. A positive first derivative of the free energy indicates the formation of a stable thin layer of the deposited material on the substrate. Otherwise, if the layer is unstable it collapses to droplets, particles, or clusters. The gold on silicon oxide system is precisely predicted by the semi-empirical and empirical models. Only the fully theoretical model fails to predict the formation of a stable thin gold layer on silicon oxide. One reason for this failure is the unknown ratio of different oxide species on the substrate surface and their unknown orientation. Therefore, an exact theoretical description of these systems is difficult. The two empirical models predict the formation of isolated droplets without a wetting layer in between. The molecular beam epitaxy (MBE) experiments show that there is no material exchange between the gold droplets formed on the silicon oxide substrate. The data indicate a re-evaporation process at elevated temperatures, which is due to a temperature-induced overall reduction of Laplace pressure in the system.

The gold on silicon system is correctly predicted by all three models at 550 °C. The first derivative of the free energy indicates that there is droplet formation above 0.34 ± 0.08 nm, which corresponds to a gold monolayer of gold. This means that an ultrathin layer of gold on silicon must be stable. This wetting layer can be considered as a mediating communication layer, or as a material transport layer, which enables Ostwald ripening processes to occur. Indeed, the MBE-formed gold droplets on silicon show material fluctuation between the droplets. Here, the increased Laplace pressure is not reduced by re-evaporation but by Ostwald ripening, which requires a medium for material transport and has a lower activation energy than evaporation. The monolayer of gold between the droplets represents this medium. In addition, the distribution of the droplet diameter values also indicates an Ostwald ripening process.

With the models shown here it is possible to predict the behaviour of droplets when the free energy is considered. This knowledge provides the understanding of dewetting phenomena of thin metal layers and how to apply these phenomena to the fabrication of nano- and microelectronic devices, such as energy conversion cells, FETs, or thermoelectric devices in which nanowires are used.

## Experimental

### Theory

The ability of a fluid to disperse or form droplets on a substrate is related to the free energy, *F*_(_*_d_*_),_ of the system, which depends on the layer thickness *d*:

[8]
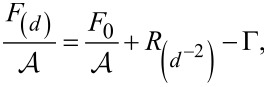


where *F*_0_ is the basic free energy, *R* is a contribution of the van der Waals energy, Γ is the so-called spreading factor and 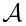 is the surface area [33]. Since *R* is a long-range force, it decreases with the square of the film thickness, *d*^2^, and increases proportionally to the difference between the Hamaker constants *A*_SF_ of solid–fluid and *A*_FF_ of fluid-fluid interactions:

[9] $$R=\left(\frac{A_{\text{SF}}-A_{\text{FF}}}{12\cdot \pi \cdot d^{2}}\right).$$

The Hamaker constants are a gauge for the interaction between particles of certain materials and the electric fields they generate [28]. This electrical responsiveness (or susceptibility) is closely related to the permittivity/polarizability, α, of the materials, the particle volume density, *m*, and the (first) ionization energies, *I,* of the particles, as Equation 7 shows:

[7] $$A_{ij}=\frac{3}{2}\cdot \pi^{2}\cdot \alpha_{i}\cdot \alpha_{j}\cdot m_{i}\cdot m_{j}\cdot \frac{\left(I_{i}\cdot I_{j}\right)}{I_{i}+I_{j}}.$$

Therefore, a fluid layer can be attracted or repelled by a substrate, leading to a wetting or dewetting tendency, respectively.

The spreading factor, Γ, is defined as the difference between the surface energy of the pristine substrate, γ_S_, and the sum of the surface energy values of the wetted substrate (γ_SF_ + γ_F_):

[10] $$\Gamma =\gamma_{\text{S}}-(\gamma_{\text{SF}}+\gamma_{\text{F}}).$$

In an ideal van der Waals system, it can be shown that the surface energy values are also associated with polarizability [34]. For real systems, however, it is more precise to use experimentally measured surface energy values that are listed in the literature for many materials. However, the determination of the surface energy between substrate and fluid, also called the interfacial energy, γ_SF_, can be more challenging. Thus the following three methods were used to determine and compare the interfacial energy values: i. The empirical method, which uses the wetting angle (the WA model), ii. the theoretical model, which uses the long-distance contribution of the van der Waals energy (the vW model), and iii. the semi-empirical model, which is in accordance with the Ewing model (the AE model).

i. The WA model: In systems in which there is droplet formation, the Young–Dupré equation [35–37] is used to determine the interfacial energy, γ_SF_. Since γ_SF_ associates γ_S_ and γ_F_ with the contact wetting angle θ, the angle between the substrate and the drop surface has to be determined:

[1] $$\gamma_{\text{SF}}=\gamma_{\text{S}}-\gamma_{\text{F}}\cdot \cos\theta .$$

However, this equation is only valid for equilibrium states on ideal surfaces. Defects on the substrate can lead to hysteresis effects that change the contact wetting angle [38]. Other surface effects, such as coarsening, ageing, or ripening can additionally lead to hysteresis effects, making the interpretation of the results even more complicated [39].

ii. The vW model: As mentioned before, the surface energy values are also connected with the polarizability of the system (i.e., with the Hamaker constants). In order to approximate the interfacial energy, it can be assumed that free surfaces with given Hamaker constants are brought together. If the surfaces reach a distance corresponding to the diameter of one particle, the nonretarded van der Waals interaction will match the ratio of the surface energy values. More precisely, the spreading factor, Γ, is the limit value of *R* at *d* = *d*_0_, where *d*_0_ is the particle diameter [34]:

[11] $$R_{\left(d_{\text{0}}\right)}=\left(\frac{A_{\text{SF}}-A_{\text{FF}}}{12\cdot \pi \cdot d_{0}^{2}}\right)=\Gamma =\gamma_{\text{S}}-\left(\gamma_{\text{SF}}+\gamma_{\text{F}}\right).$$

Thus the interfacial energy can be determined by the *R* and surface energy values, according to:

[2] $$\gamma_{\text{SF}}=\left(\gamma_{\text{S}}-\gamma_{\text{F}}\right)-\left(\frac{A_{\text{SF}}-A_{\text{FF}}}{12\cdot \pi \cdot d_{0}^{2}}\right).$$

iii. The AE model: The AE model consists of a semi-empirical alternative to determine the interfacial energy, γ_SF_ [40]. R. H. Ewing postulated that the interfacial energy of a solid material and its melted form consists of an energetic part of the solid, γ_SF_^S^, and an energetic part of the fluid, γ_SF_^F^, given that the chemical and physical bonds between the solid material and its melted form coexist in the system (for more information about R. H. Ewing's interfacial energy model, please see [41–42]). Therefore, the interfacial energy based on the AE model is given by:

[3] $$\gamma_{\text{SF}}=\gamma_{\text{SF}}{}^{\text{S}}+\gamma_{\text{SF}}{}^{\text{F}}.$$

Here, the model was not used to calculate the interfacial energy between a solid and its own melted material; instead, it was used to determine the interfacial energy between a solid and a fluid from another compound. This approach shows results that are in a good approximation with the actual value of γ_SF_.

However, the wettability of a system is not directly predictable by the free energy itself, but by the slope of its function. Accordingly, this is given by the first derivative of the free energy. In order to derive the free energy, a constant volume for the system with *A* = *V*·*d* needs to be considered. For the WA model, the interfacial energy is given by:

[4] $$\frac{\partial \frac{\Delta F_{\left(d\right)}}{\Delta V}}{\partial d}=-\left(\frac{A_{\text{SF}}-A_{\text{FF}}}{4\cdot \pi \cdot d^{4}}\right)-\frac{\gamma_{\text{F}}\left(\cos\theta +1\right)}{d^{2}},$$

whereas for the vW model the interfacial energy is given by:

[5] $$\frac{\partial \frac{\Delta F_{\left(d\right)}}{\Delta V}}{\partial d}=-\left(\frac{A_{\text{SF}}-A_{\text{FF}}}{4\cdot \pi \cdot d^{4}}\right)+\frac{A_{\text{SF}}-A_{\text{FF}}}{12\cdot \pi \cdot d_{0}^{2}\cdot d^{2}},$$

and for the AE model, the interfacial energy is given by:

[6]
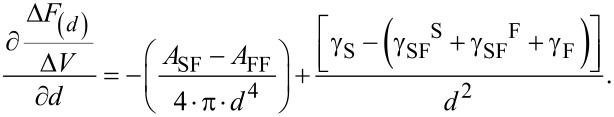


If the first derivative of the free energy is positive, then the fluid wets the substrate. In contrast, for negative values, dewetting occurs. If the first derivative of the free energy is zero, there is droplet formation if a maximum stable thickness is exceeded. In this case, a material layer with the maximum stable thickness (i.e., the wetting layer) may persist between the droplets. However, all the calculations shown here apply only to thickness values below the capillary length (>1 µm) of the fluid, since gravity can be neglected within this range [34].

Table S1 (Supporting Information File 1) shows the set of values required for calculating the free energy per unit area and its first derivative for gold on silicon oxide and gold on silicon.

### Experiments

The UHV−PVD experiments were carried out in a MBE chamber with a basal pressure of 2 × 10^−10^ mbar. Two different substrates were used for these experiments: The first substrate was single-crystalline Si(111) wafers of 25 × 25 × 0.525 mm^3^ in size. These wafers where chemically cleaned by the conventional Radio Corporation of America (RCA) etching processes, known as RCA 1 and RCA 2, to get hydrophilic silicon surfaces immediately before transferring to the UHV system. Before the growth of the nanowires, the Si(111) substrates were annealed at 900 °C for 30 min to desorb residual silicon oxide from the surface. The second substrate consisted of a chemical vapour deposited SiO*_x_* layer (thickness of 500 nm) on top of a Si(001) wafer. The subsequent chemical–mechanical polishing provided a highly flat surface (RMS = 0.3 nm). Gold with a nominal thickness of 1 nm was evaporated from an effusion cell onto the heated substrate with a deposition rate of 0.01 nm/s, which was calibrated before with an Inficon XTC/3 quartz crystal microbalance (Bad Ragaz, Switzerland). Series of experiments were carried out at substrate temperatures of 300, 400, 500, 550, 600, and 650 °C, in case of SiO*_x_* on Si(001), and substrate temperatures in the range of 400 to 700 °C, in steps of 50 K, for Si(111) wafers. The gold droplet formation was analysed with a dual-beam FEI Nova 600 NanoLab scanning electron microscope (Thermo Fisher Scientific, MA, USA). The SEM images were used to determine shape, size, number and wetting angles of the gold droplets. The growth of germanium structures occurred at temperatures of 500 °C and at germanium deposition rates of 0.005 nm·s^−1^. Since the solubility of germanium in gold is high, a supersaturated mixture will form. To reduce the supersaturation, germanium precipitates at the bottom of the gold droplet. This way, germanium nanowires may grow. This process is known as vapour–liquid–solid process (VLS).

## Supporting Information

File 1 Parameters used to obtain the free energy per unit area for Au/Si and Au/SiO*_x_* substrates.
