# Supplementary material for: Analysis of catalyst surface wetting: the early stage of epitaxial germanium nanowire growth
Source: Beilstein J Nanotechnol. 2020 Sep 9;11:1371–80. doi: 10.3762/bjnano.11.121 (PMC7492698; doi:10.3762/bjnano.11.121)
Supplement: File 1 — Parameters used to obtain the free energy per unit area for Au/Si and Au/SiOx substrates. [file Beilstein_J_Nanotechnol-11-1371-s001.pdf]

BEILSTEIN JOURNAL OF NANOTECHNOLOGY

# Supporting Information

for

# Analysis of catalyst surface wetting: the early stage of epitaxial germanium nanowire growth

Owen C. Ernst, Felix Lange, David Uebel, Thomas Teubner and Torsten Boeck

*Beilstein J. Nanotechnol.* **2020,** *11,* 1371–1380. doi:10.3762/bjnano.11.121

# Parameters used to obtain the free energy per unit area for Au/Si and Au/$SiO_x$ substrates

License and Terms: This is a supporting information file under the terms of the Creative Commons Attribution License (https://creativecommons.org/licenses/by/4.0). Please note that the reuse, redistribution and reproduction in particular requires that the authors and source are credited.
The license is subject to the *Beilstein Journal of Nanotechnology* terms and conditions: (https://www.beilstein-journals.org/bjnano)

**Table S1:** Required values for the theoretical models and the resulting interfacial energies by wetting angle, by van der Waals energies and according to R. H. Ewing.

| | **RT (25 °C)** | | | **550 °C** | | |
|---|---|---|---|---|---|---|
| | **Silicon oxide** | **Gold** | **Silicon** | **Silicon oxide** | **Gold** | **Silicon** |
| **Hamaker constant $A_{ij}$ [$10^{-19}$ J]**[1–9] | **3.08–9.96** | | | **3.08–9.96** | | |
| | | **2.90–6.43** | | | **2.90–6.43** | |
| | | **2.81–5.29** | | | **2.81–5.29** | |
| **Surface energy [J/m²]** | **0.01** [10] | **1.26** [11] | **1.23** [12] | **0.01** [10] | **1.22** [11] | **1.23** [12] |
| **Wetting angle** | 84° | | | 84° | | |
| | | 42° | | | 42° | |
| **energetic portion in interfacial energy** [13] | 0.155 | −0.018 | 0.286 | 0.155 | −0.050 | 0.285 |
| **Particle diameter [nm]** | | 0.35 [14] | | | 0.35 [14] | |
| **Interfacial energy WA [J/m²]** | **−0.1** | | | **−0.1** | | |
| | | **1.1** | | | **1.1** | |
| **Interfacial energy VW[J/m²]** | **−1.35** | | | **−1.27** | | |
| | | **0.09** | | | **0.16** | |
| **Interfacial energy AE [J/m²]** | **0.14** | | | **0.10** | | |
| | | **0.27** | | | **0.24** | |

## References


1. Krupp, H. *Adv. Colloid Interface Sci.* **1967**, *1* (2), 111–239

2. Visser, J. *Adv. Colloid Interface Sci.* **1972**, *3* (4), 331–363

3. Schwerdtfeger, P. *Cent. Theor. Chem. Physics, Massey Univ.* **2015**

4. Buttner, F. H.; Udin, H.; Wulff, J. *JOM* **1951**, *3* (12), 1209–1211

5. Paradisa, P. F.; Ishikawaa, T.; Koikeb, N. *Gold Bull.* **2008**, *41* (3), 242–245

6. Kim, J. Y.; Hwang, M. S.; Kim, Y.-H.; Kim, H. J.; Lee, Y. *J. Appl. Phys.* **2001**, *90* (5), 2469–2473

7. Bettin, H.; Toth, H. *IEEE Trans. Instrum. Meas.* **2007**, *56* (2), 257–261

8. Biggs, S.; Mulvaney, P. *J. Chem. Phys.* **1994**, *100* (11), 8501–8505

9. Bergström, L. *Adv. Colloid Interface Sci.* **1997**, *70*, 125–169

10. Janssen, D.; De Palma, R.; Verlaak, S.; Heremans, P.; Dehaen, W. *Thin Solid Films* **2006**, *515* (4), 1433–1438

11. Egry, I.; Lohoefer, G.; Jacobs, G. *Phys. Rev. Lett.* **1995**, *75* (22), 4043

12. Jaccodine, R. J. *J. Electrochem. Soc.* **1963**, *110* (6), 524–527

13. Jones, H. *Mater. Lett.* **2002**, *53* (4–5), 364–366

14. Clementi, E.; Raimondi, D. L.; Reinhardt, W. P. *J. Chem. Phys.* **1967**, *47* (4), 1300–1307
